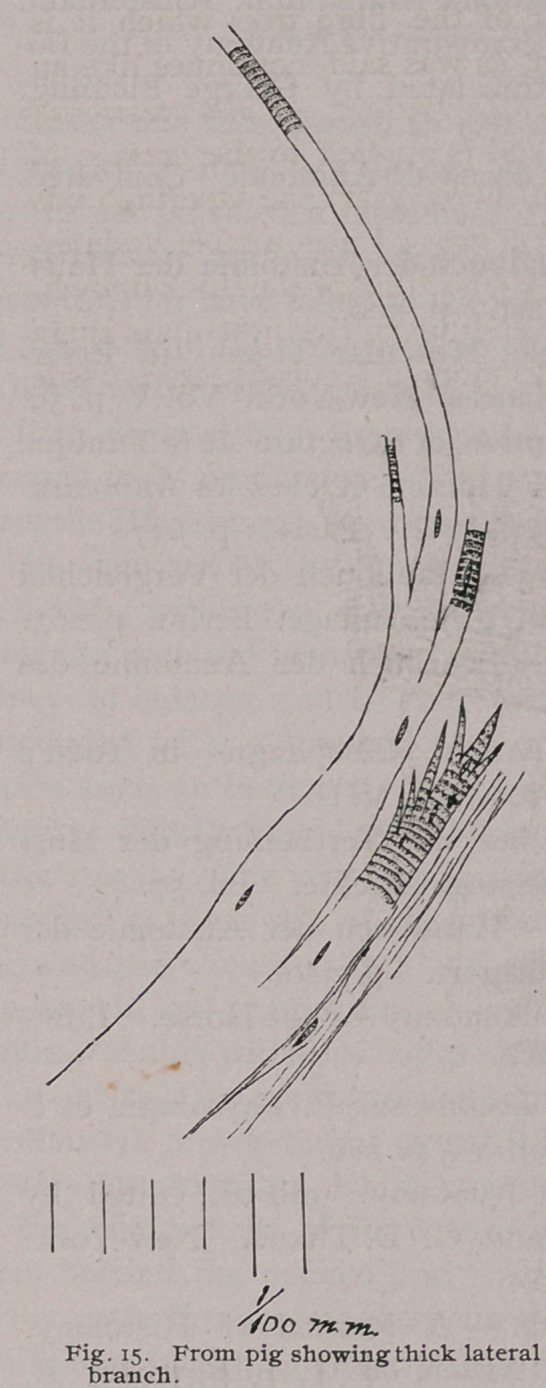# The Muscular Coats of the Œsophagus of the Domesticated Animals

**Published:** 1889-01

**Authors:** Leonard Pearson

**Affiliations:** Cornell University


### ART. VI.—THE MUSCULAR COATS OF THE ŒSOPHAGUS OF THE DOMESTICATED ANIMALS.

BY LEONARD PEARSON, B. S.,
*Cornell University.*

HISTORICAL SKETCH.—Human anatomists are agreed that in man the muscular coat of the œsophagus is composed of two or more less regular and distinct layers, *i. e.*, an external longitudinal and an internal circular. The longitudinal layer is described as originating from the ridge on the dorsal face of the cricoid cartilage, and from the inferior constrictor of the pharynx, while the circular layer comes from the inferior of the pharynx alone.

From Gillette (7),[1] we find that these layers are not as regular as would be inferred from the descriptions. In the pharyngeal part of the œsophagus, the longitudinal layer is thinner and more uniform than in the gastric part, where it is somewhat separted into bundles, and of these "some are parallel, some inter-cross, some divide and anastomose with each other. This inter-crossing and anastomosing takes place not only superficially, but also below the surface; that is to say, the ectal fibres become ental, and *vice versa*. In certain places a complete and almost inextricable entanglement is found." With respect to the circular layer, the same author says that the fibres are not arranged in a regular manner. "There are complete and incomplete rings, sometimes inter-crossing at a more or less acute angle, and enclosing between them fibres that connect one ring with another."

The veterinary anatomists, presumably following the human anatomists, for a long time described a similar arrangement of the œsophageal muscles in the domestic animals. Geradus Blasius (2) in 1681 described the arrangement of the muscle fibres in the œsophagus of the dog as follows: "The fibres are divided into two spirals, which meet in definite places, on the anterior and posterior walls, and intersect each other so that one goes under the other. They intersect by turn, so that the right seeks the left and

[1] The number in parenthesis following au author's name refers to the bibliography at the end of this paper.

the left the right. What constituted the external tunic becomes the internal, and again the external." This arrangement, described by Blasius, is not found mentioned by any other author.

Leyh (12), in 1859, said that there were two muscular coats of the œsophagus; an external longitudinal, and an internal circular. He did not mention any difference as existing between the horse and the ox.

Strangeways (17), 1870, also divided the muscular coat into two layers, the external being composed of longitudinal fibres, and the inner of "spiral or circular" fibres.

Chauveau (3), in 1872, makes it evident that these coats are not entirely distinct, as he says that toward the inferior extremity of the canal they inter-cross in an almost inextricable manner.

In 1868, Klein (11) described an exceedingly complex arrangement of the fibres in the dog. In the pharyngeal eighth, he said, there is an external longitudinal and an internal circular layer; in the next two-eighths the fibres of both layers decussate obliquely and at right angles; in the next three eights there is an inner longitudinal and an external circular layer; in the next eighth there are three layers: an internal longitudinal, a middle circular and an external longitudinal, the last mentioned layer, being derived from the other two. In the last, or gastric eighth, there are also three layers: an internal oblique, a middle transverse and an external longitudinal. Klein does not say how these results were obtained, but from the figure it is evident that his conclusions were drawn from the study of transections.

Gillette (7) made a study of the "muscular tunic of the œsophagus in the animal series," and these briefly were his conclusions: In the dog there is no longitudinal layer. The superficial or ectal layer is composed of circular or elliptical fibres that cross on the dorsal wall in such a way as to represent a raphe. The second layer is subadjacent to the first, and is composed of fibres that have a direction opposite to the first, which they cross at an acute angle. A third very thin coat is mentioned. In the cat there are two layers, and the longitudinal arrangement is shown only in the caudal or gastric end. In the sheep there is no longitudinal layer; and the muscular tunic is composed of an entanglement of fibres that present no regularity. In the ox the arrangement is similar to that found in the sheep. In the horse there are two thick longitudinal bands of fibres extending one on each side, from the pharynx to the junction of the middle and gastric thirds. Between these longitudinal bundles, and springing from them are annular bundles that cross at a "raphe" on the dorsal wall and become mixed on the ventral wall. In the caudal third the regularity has disappeared. Two internal columns were seen, corresponding to those on the surface.

Franck (5), in 1883, described another system as existing in the horse. He says, in substance, that there are two lateral bundles, as above, and from these the oblique spirals take their origin. Upon the ventral and dorsal wall is a scarcely noticeable seam where the spirals come together. This constitutes the ectal layer. The ental layer is thinner, and its spirals cross those of the ectal at an acute angle. In ruminants the muscular arrangement is in reality as it is in the horse, but no longitudinal bands are present. There are two layers of flat spirals, which cross at an acute angle.

Both Franck and Gurlt (8) call attention to the fact that fat is frequently deposited between these spirals.

ARRANGEMENT OF THE FIBRES.[1] *Horse.*—The muscular coat of the œsopagus conmences in the horse at the caudal part of the pharynx, by two small bundles given off from the inferior constrictor of the pharynx, and by two bands arising from the caudal border of the arytenoid cartilage (authors). From these fibres two layers are formed, but they cannot be styled ectal and ental, as fibres that are at one place ectal are at another place ental and *vice versa.* The fibres of the two layers form two spirals, which run in opposite directions around the œsophagus. Upon the dorsal and ventral walls the two spirals decussate. Those fibres that were ectal up to this time become ental, and the ental become ectal. It is, therefore, evident that in each passage around the œsophagus one-half of the length of the spirals are ectal and the other half ental. On the ventral wall the spirals, in meeting, form an angle with the apex pointing toward the stomach, while on the dorsal wall the apex, of necessity, points towards the pharynx.

Some of the fibres that are ectal unite on the ventral and dorsal aspects to form two longitudlnal bands that extend from the pharynx two-thirds of the distance to the stomach. At this point their fibres become lost in the spirals, which gradually becomes less regular, until the typical spiral arrangement can be seen in but a few places. The longitudinal bands do not remain on the

[1] The following discussion does not refer to the *Muscularis muscosæ*.

meson and cover the line of decussation at all points, but in places becomes more lateral.

*Swine.*—In the hog the muscular coat is also made up of two spiral layers, which decussate upon the ventral and the dorsal walls. (Figs. 1 and 2). The line of decussation is quite distinct upon the dorsal wall for the first one-third. On the ventral wall for the same distance, the place of crossing shows only in part, as some of the fibres do not cross at this point, and by passing over, the line of decussation is hid from view. By removing these fibres, the crossing place can be plainly seen. Caudad of the first one-third until 8–12 cm. from the stomach, some of the fibres become very much inclined, and extend longitudinally along the dorsal and ventral aspects of the œsophagus, covering the line of decussation. As before, this can be seen by removing the superficial fibres. In the 8–12 cm. next to the stomach, the ectal fibres become more nearly perpendicular to the longitudinal axis of the œsophagus, while the ental fibres retain the former inclination of about 45°.

Fig. 2. Fig. 1.

Fig. 1. Dorsal aspect of the œsophagus of a large pig. X ½.

Fig. 2. Ventral aspect of same.

*Sheep.*—In the sheep, where regurgitation is a normal and frequent act, it might be supposed that there would be a well-developed longitudinal layer of muscle, but such is not the case. Even the longitudinal bands found in the horse and hog are not present. The muscular fibres form two oppositely directed spirals, which cross one another on the dorsal and ventral walls. Both lines of decussation are quite well marked. Throughout the length of the tube the ectal fibres are only slightly inclined, but as soon as they become ental, their angle of inclination increases in the cephalic third to 65°, decreasing to 45° in the middle third, after which the ental and ectal fibres assume approximately the same angle with the meson—about 45°—and thus meet each other at a right angle. In the last 2–5 cm. the ectal fibres become almost longitudinal before extending upon the stomach.

Fig. 3

Dorsal aspect of œsophagus of cow 25 cm. caudad of pharynx. Ectal fibres removed. Cephalad of a. X I.

At many places in the length of the œsophagus small offshoots take their origin from an otherwise regular bundle of fibres. (See Fig. 4.) These offshoots pursue an irregular course, sometimes extending longitudinally for a few centimeters, and then dipping down and continuing in the regular course (Fig. 4, *a*), or they may simply be inclined at a different angle from the other fibres of the layers (Fig 4, *b*.)

*Ox.*—The disposition of the muscular fibres in this animal is very similar to the arrangement found in the sheep, but there are some constant differences. In the pharyngeal third it looks as though the fibres regularly encircled the œsophagus, but by removing the superficial layers upon the dorsal and ventral walls, the lines of decussation can be seen. The ental fibres in the pharyngeal third have an inclination from the longitudinal axis of about 30°. As soon, however, as they cross and become ectal, they assume a direction nearly perpendicular to the axis (Fig. 3). As the

Fig. 4

Ventral œsophagus of steer XI. To show irregularities.

ectal fibres approach the line of decussation, some, instead of passing under the bands from the other layer, pass over, and thus coveri the crossing place. It is to this fact that the annular appearance

Fig. 5. Dorsal aspect of œsophagus of large dog x ½.

Fig. 6. Ventral aspect of same.

is due. Continuing toward the stomach, the ectal fibres become less inclined to the axis, while the inclination of the ental fibres increase, so that at the junction of the middle and gastric thirds, each layer has an inclination of about 45°, and thus continue to the stomach.

As with the sheep, there are irregularities in places, and these are much of the same nature. Longitudinal bands may extend along the surface for a few centimetres, or some fibres may take an inclination differing from that of the spirals (Fig. 4, *a. b.*) None of these offshoots extend far, and if they belong to a regular system the connection was not discovered.

*Dog.*—In the dog the arrangement of the muscular fibres is more regular and uniform throughout the length of the tube than in any other animal examined. As before, two spirals are found that decussate upon the dorsal and ventral walls. (Figs. 5 and 6.) The lines of decussation are distinct for their entire lengths. At the beginning of the œsophagus the ectal fibres have an inclination of about 72° to the longitudinal axis, but upon crossing the line of decussation and becoming ental, they turn cephalad or caudad at such an angle as to make their direction at right angles to that of the fibres covering them. The directions of the fibres gradually become equalized until both have an inclination of 45°.

There are at the gastric end a few small bundles following a course differing more or less from the regular one. These bundles all pass to the stomach, and are the only signs of irregularity in the tube. If transections were made at this point, and descriptions based upon them alone, it would be easy to repeat Kline's error and say that at the caudal end of the œsophagus there are three layers of muscular tissue. The so-called third layer exists only where a bundle of fibres pursues an erratic course.

*Cat.*—In this animal the spiral arrangement is again found, but not in the entire length of the tube. The ectal and ental fibers first cross at an angle of 150°, which gradually decreases to 90°. This decrease is occasioned by the ectal fibres becoming more nearly longitudinal and the ental more nearly circular.

The circular layer is not spiral in character, nor are the fibres grouped into distinct rings, but it is a uniform sheet encircling the caudal part of the tube. In the caudal one-third, there is no crossing of fibres, which ceases, of course, when the two spirals become distinct from each other.

The œsophagus of the cat is peculiar, in that the longitudinal and circular plan is more largely developed than in the other

Fig. 7

Fig. 8

Fig. 9

Fig. 10.

**Diagrams showing typical arrangements of fibers in domestic animals.**

**Figs. 7–8.—Ventral and dorsal aspects ectal layer complete.**

**Figs. 9–10.—Ventral and dorsal aspects with ectal layer removed to show the ectal layer.**

domestic animals, In the sheep this arrangement also exists in the gastrict end of the tube, but for a shorter distance.

SUMMARY OF ARRANGEMENT OF FIBRES.—1. The muscular coat of the œsophagus is divided into two layers.

2. These layers cannot be designated longitudinal and circular.

3. These layers cannot be designated ectal and ental.

4. Fibres that are on one side ectal are on the other ental, and *vice versa.*

5. If longitudinal and circular layers exist with any regularity, it is only in the gastric end of the tube.

6. The typical arrangement may be thus described (see Figs. 7 to 10): In the walls of the œsophagus there are two layers of muscle fibres, arranged in spirals. These spirals are wound in opposite directions around the tube. Upon the dorsal and ventral walls they cross each other, by dividing into small bundles which interdigitate, the ectal becoming ental, and the ental, ectal, This can be nicely represented by folding the hands thus:

MICROSCOPIC ANATOMY.*—The first feature that impresses one upon making a microscopic examination of the muscle of the œsophagus is, that in some of the domesticated animals a part of the tissue is composed of striated, and a part of unstriated fibres. In brief, the relations of the two kinds of muscles are as follows:

In the horse, non-striated fibres begin with the gastric third and increase in number approaching the stomach, but a few striated fibres continue to the stomach.†

In the pig, non-striated fibres begin to appear 12–18 cm. from the gastric extremity, and increase in number to the stomach, but, as before, some striated fibres continue to the stomach, and in this case are much more abundant.

In the sheep, ox and dog, no non-striated fibres are found cephalad of the diaphragm.

---

*The following discussion does not refer to the *muscular misuscosæ.*

†This was true of the only horse's œsophagus examined microscopically.

In the cat, when the ectal fibres first become longitudinal, the non-striated fibres begin to appear, and soon they alone are found. In the ental or circular portion, the non-striated fibres appear at the same level and sooner exclude the striated.

TERMINATIONS OF FIBRES. In every œsophagus examined, fibres gradually tapering to a point were found. They were present in nearly all of the preparations examined, showing this to be an exceedingly common method of ending. Near the end of the fibre there is a marked swelling, and at this point a large nucleus. Just before making this expansion, the striæ fade away, and the material in which the nucleus is imbedded, as well as the fibre beyond, is clear, except a few granules (Fig. 11.)

Fig. 11.—Termination of a fiber from the sheep.

In the sheep, ox, dog and pig, fibres frequently taper to within 2 mm. from the end, when lateral branches are given off at intervals for the rest of the distance. Sometimes there is but one such branch, and sometimes as many as five (ox.) These branches may be given off before the striæ cease, in which case they are striated themselves, and end in a swelling and nucleus, as above, or they may be given off at the swelling, when they resemble in appearance the tip of the fibre (Fig. 12.) In the latter case the branches are very short. These tapering ends are applied to another fibre at a place where it is of full size (6). The end seems to be retained in this position by a sort of cell cement. No connective tissue has been noted adhering to an end of this kind.

In the ox, there is a kind of ending where, although the fiber tapers, it does not come to a point. When a diameter of about .01 mm. is reached, a blunt end is formed, and connective tissue fibres extend from the ends and sides of the ending along the side of an adjacent full-size fibre for a distance of about .2 mm. (Fig. 13). This condition was seen distinctly in but two cases.

Fig. 12. Termination of a fiber from the dog. Shows branch and mode of union.

In the pig only, were fibres found to end without first becoming narrower, although tapering ends were also plenty. These endings are of two kinds: In the first case, the branches are all near the end, and may all be considered as terminal (Fig. 14), and in the second case there is a thick lateral branch, after which the fibre tapers and gives off small branches as an ordinary tapering end (Fig. 15.)

In the first case, the condition may be compared to the end of a tree trunk that has been blown down and is shivered at the point of fracture. At the end of the fibre are two to six branches that separate into so many small branches as to resemble a brush. While all the primary branches may not arise from the end of the fibre, they spring from the sides very near to the end. The primary branches are striated, and the striæ show for part of the length of the secondary branches, when they fade out, leaving the apex clear. How a fibre of this sort is attached to another I was unable to determine. It does not at all seem likely that such a thick end would be an overlapping end, and nothing was seen to favor this view. All of the endings of this character found were surrounded by more or less connective tissue.

In the second class a large branch is given off .15 to .2 mm. from the end of the fibre. This branch is short and simple, and is divided at the end like the fibres of the first-class. The branch is sometimes of half of the diameter of the fibre from which it is given off. The remaining portion, as was said, continues like an ordinary tapering end of the branching variety. This branching tapering end is applied to the surface of a full-sized fibre, as are the tapering ends described above. I was unable to determine the connections of the thick branch. Like the endings of the first class there was always more or less connective tissue

Fig. 13. Termination of a fiber from the ox. Shows blunt end and mode of union.

Fig. 14. Brush like termination from pig.

adhering to its end and sides. Where the striated and unstriated fibres join, the former terminate in an unbranched, tapering end that is surronnded by unstriated muscle cells joined to it by means of cell cement (6.)

METHODS.—To soften and remove the connective tissue so that e muscle fibres could be easily separated, three methods were employed :—

Fig. 15. From pig showing thick lateral branch.

1. Boiling inwater.

2. Macerating in 20 per cent. nitric acid. The œsophagus should be filled with the acid and the ends tied, then suspended in a long jar of the same liquid. It is necessary to allow it to remain thus from six hours to three days, depending upon the temperature.

3. Heating in 5 to 10 per cent. nitric acid ($H N O_3$). This method is used when it is desired to study the specimen at once.

The first method was useful only in the gross anatomy, and even in this case was not as satisfactory as the methods following. Boiling enough to make the fibres easily separable sometimes renders them friable. If the second or third method is used, the material must be perfectly fresh, for it is found that otherwise the muscular fibres will soften before the connective tissue. After treatment with nitric acid the muscle was found to continue to soften if kept in water or alcohol. Prof. S. H. Gage found that this softening could be prevented by keeping the tissue in a saturated aqueous solution of alum. Before the immersion in alum water, the fibres cannot be satisfactorily stained, but after remaining in this solution for a few hours, hæmatoxylon stains them excellently.

### BIBLOGRAPHY.

1. *Allen, Harrison, 1884.*—A system of Human Anatomy. Philadalphia. p. 641.

2. *Blasius Gerardus, 1681.*–Anatome Animalium. Amsterdam.

3. *Chauveau, A, 1873.*—The Comparative Anatomy of the Domesticated Animals. 2nd ed., translated by George Fleming. New York. p. 379.

4. *Cuvier, Georges, 1835.*—Lecons d' Anatomie Compareé. Paris. Vol. IV, p. 15.

5. *Franck, Ludw., 1883.*—Handbuch der Anatomie der Hausthiere. Zweite Auflage. Stuttgart. p. 500.

6. *Gage, S. H., 1887.*—Article, Muscular Tissue, in Reference Hand-Book of the Medical Sciences. New York. Vol. V, p. 59.

7. *Gillette, Dr., 1872.*—Description et Structure de la Tunique Musculaire de l' œsophage chez l' Homme et chez les Animaux. *Journal de l' Anatomie et de la Physiologie.* Paris. p. 617.

8. *Gurlt, Ernst Fredrick, 1873.*—Handbuch der Vergleichen der Aatomie der Haus-Säugethiere. Fifte Auflage. Berlin. p. 263.

9. *Hoffman, C. E. E., 1877.*—Lehrbuch der Anatomie des Menchen. Erlangen. p. 543.

10. *Johnson, George, 1845.*—Article, Œsophagus, in Todd's Cyclopædia of Anatomy. London. Vol. VIII.

11. *Klein, Emanuel, 1868.*—Uber die Vertheilung der Muskeln des Œsophagus. Weiner Sitzungsberichte. Bd. 57.

12. *Leyh, Friedrich A., 1859.*—Handbuch der Anatomie der Haustiere. Zweite Auflage. Stuttgart. p. 380.

13. *M' Fadyean, J, 1884.*—The Anatomy of the Horse. Edinburgh. p. 150.

14. *Milne, Edward H., 18–.*—Lecons sur la Physologie et l' Anatomie Compareé. Paris. Vol. VI, p. 280.

15. *Quain, 1882.*—Elements of Anatomy. 9th ed., edited by Allen Thompson, E. H. Shafer and G. D. Thane. New York. Vol. II, p. 586.

16. *Stricker, S., (editor), 1872.* — A Manual of Histology. Translated by Henry Power. (Article on Œsophagus, by E. Klein). New York.

17. *Strangeway, 1870.*—Veterinary Anatomy. 2d ed., edited by I Vaughn. New York.

## Figures and Tables

**Fig. 2. Fig. 1. f1:**
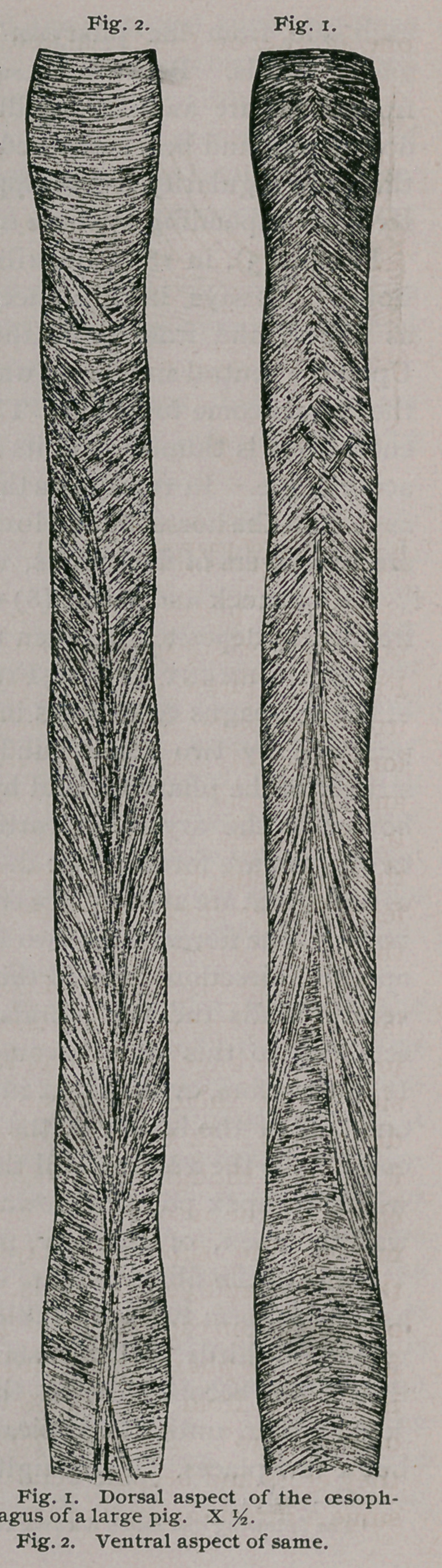


**Fig. 3 f2:**
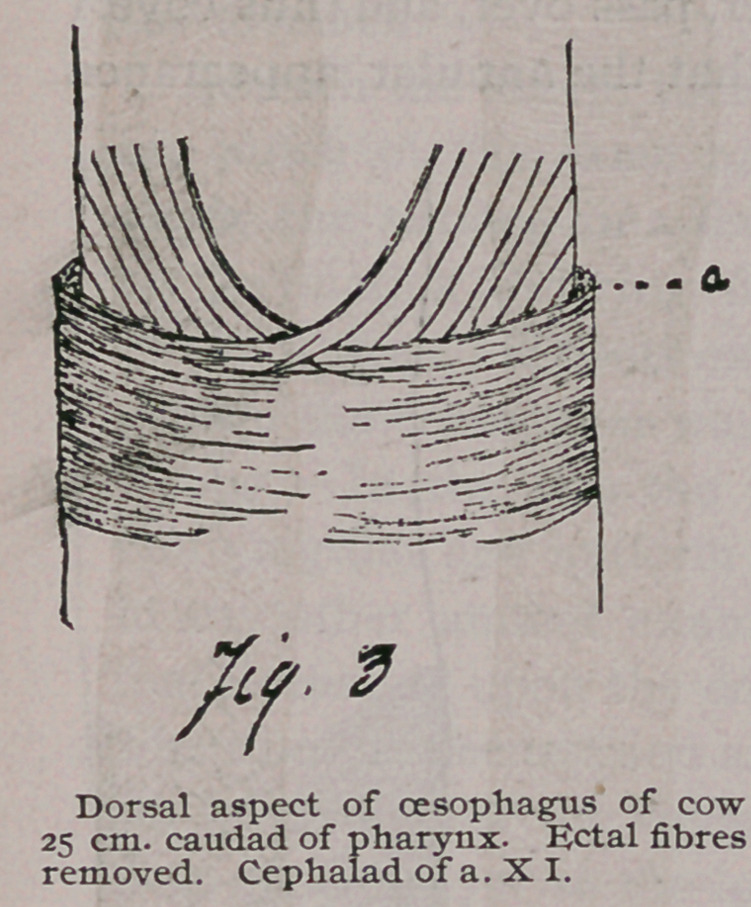


**Fig. 4 f3:**
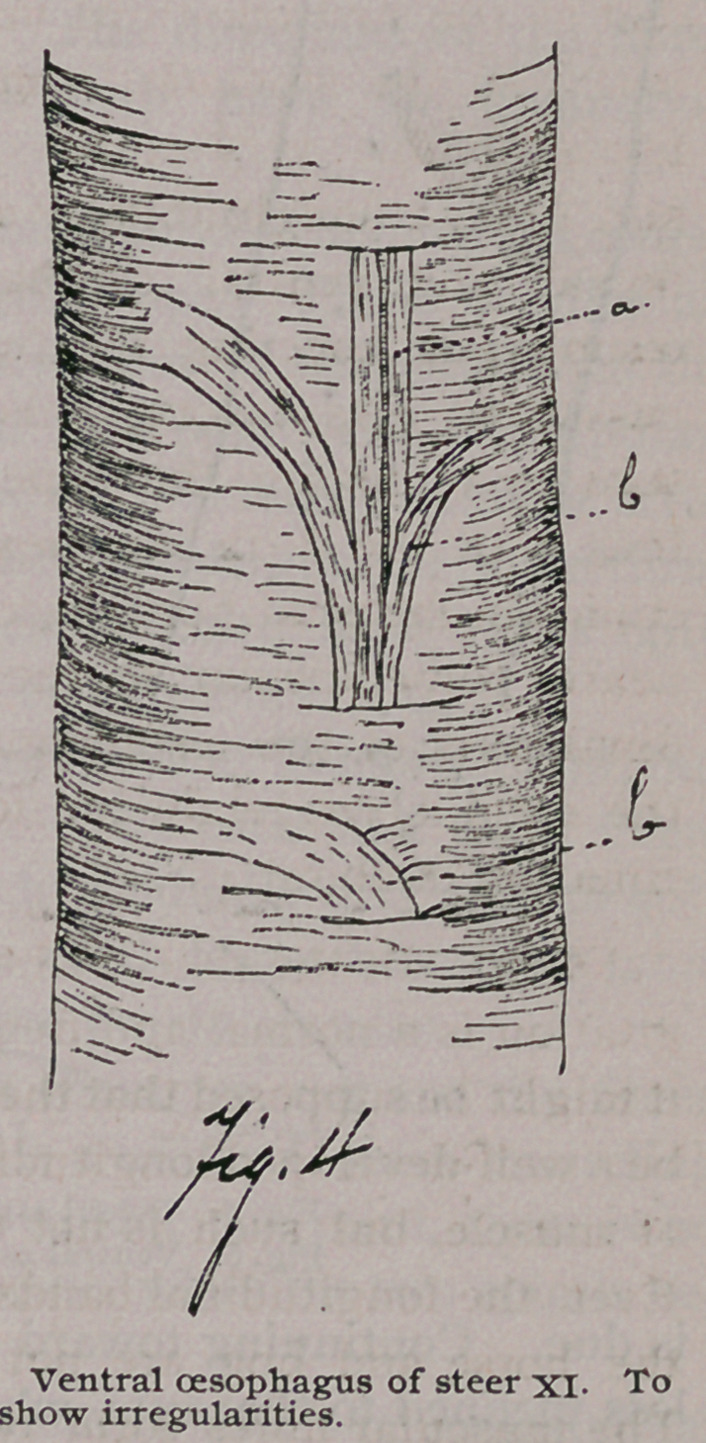


**Fig. 5 Fig. 6 f4:**
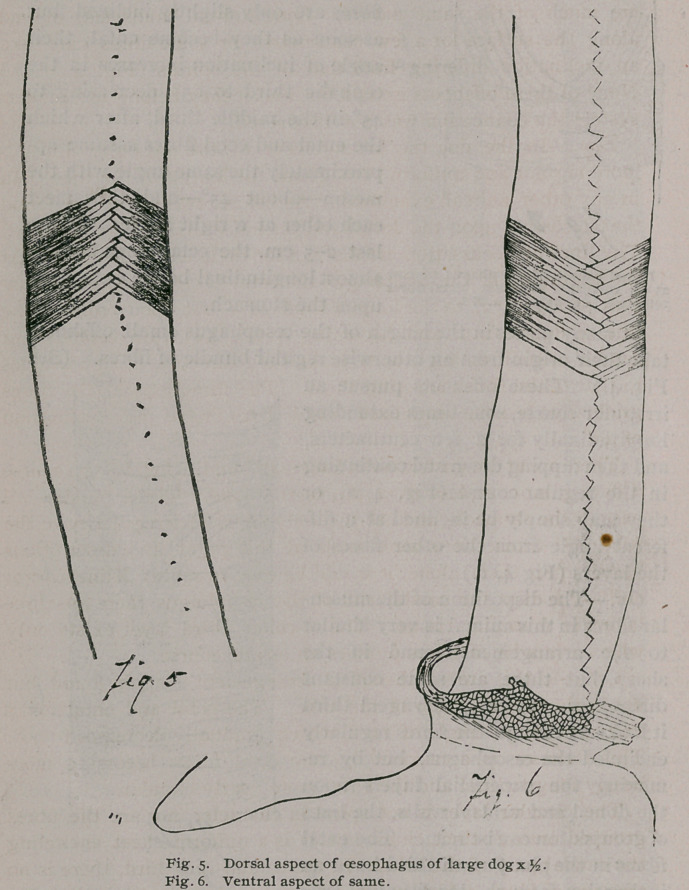


**Fig. 7 Fig. 8 Fig. 9 Fig. 10 f5:**
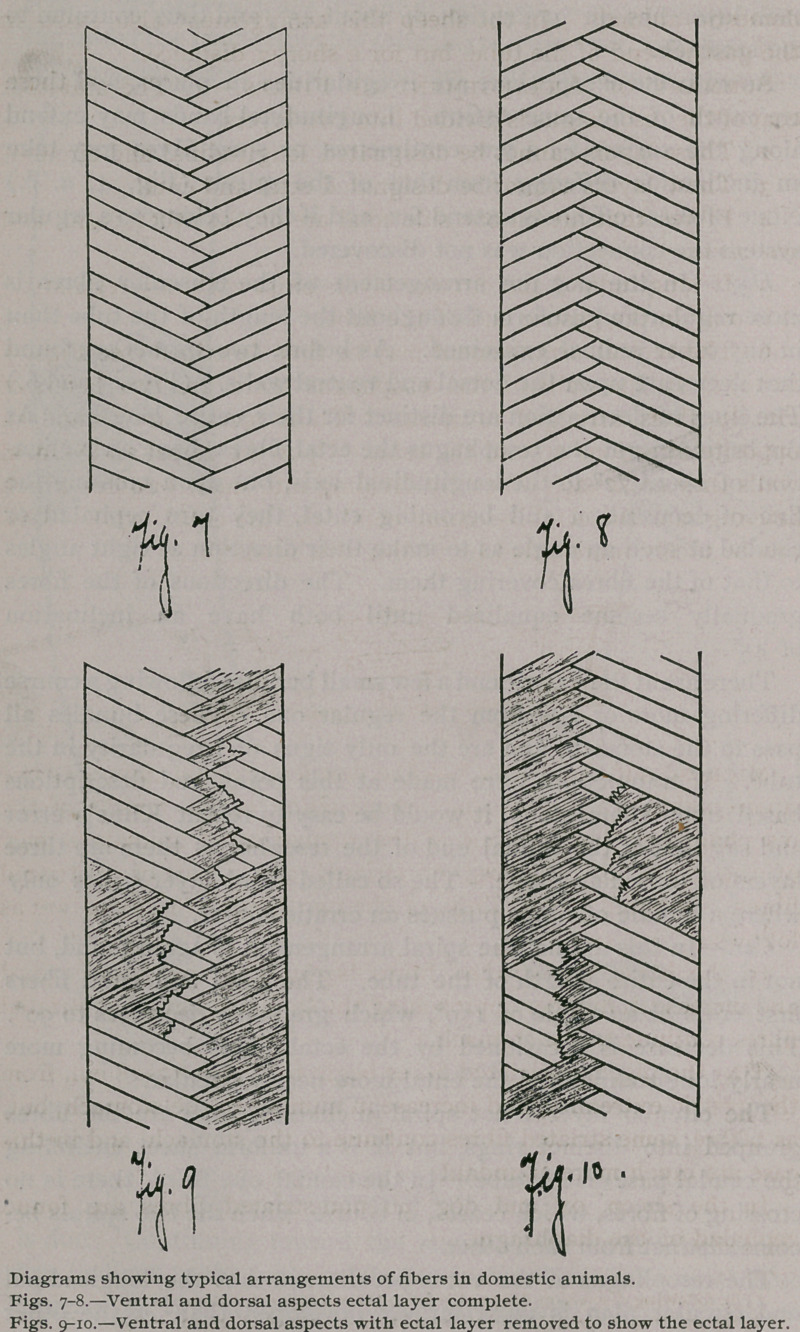


**Figure f6:**
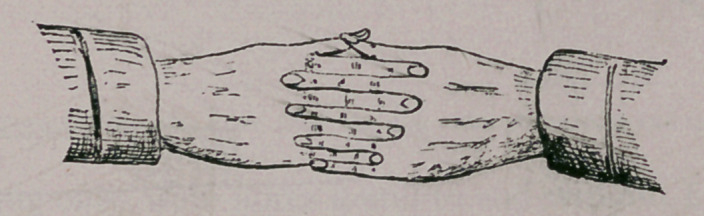


**Fig. 11. f7:**
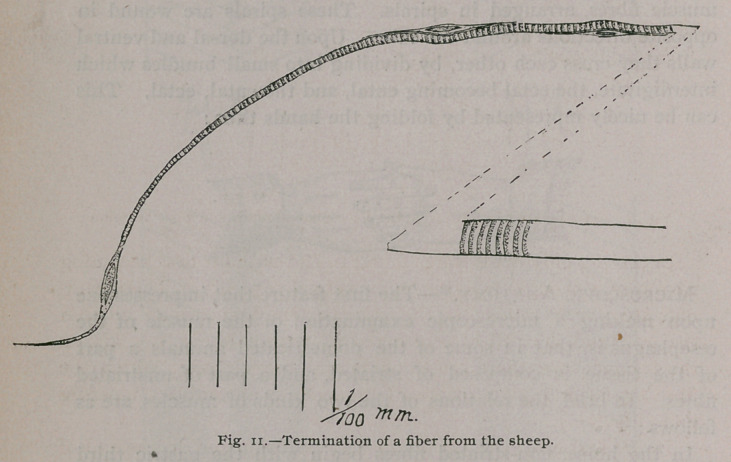


**Fig. 12. f8:**
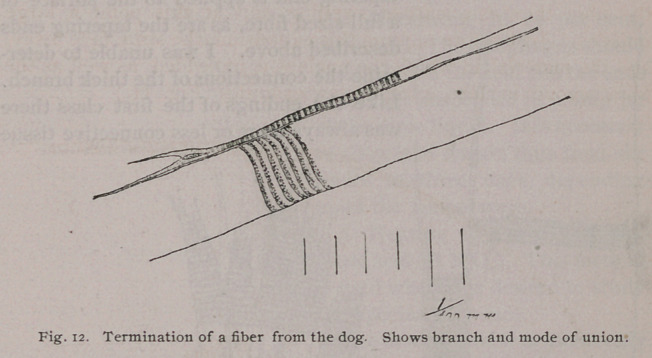


**Fig. 13. f9:**
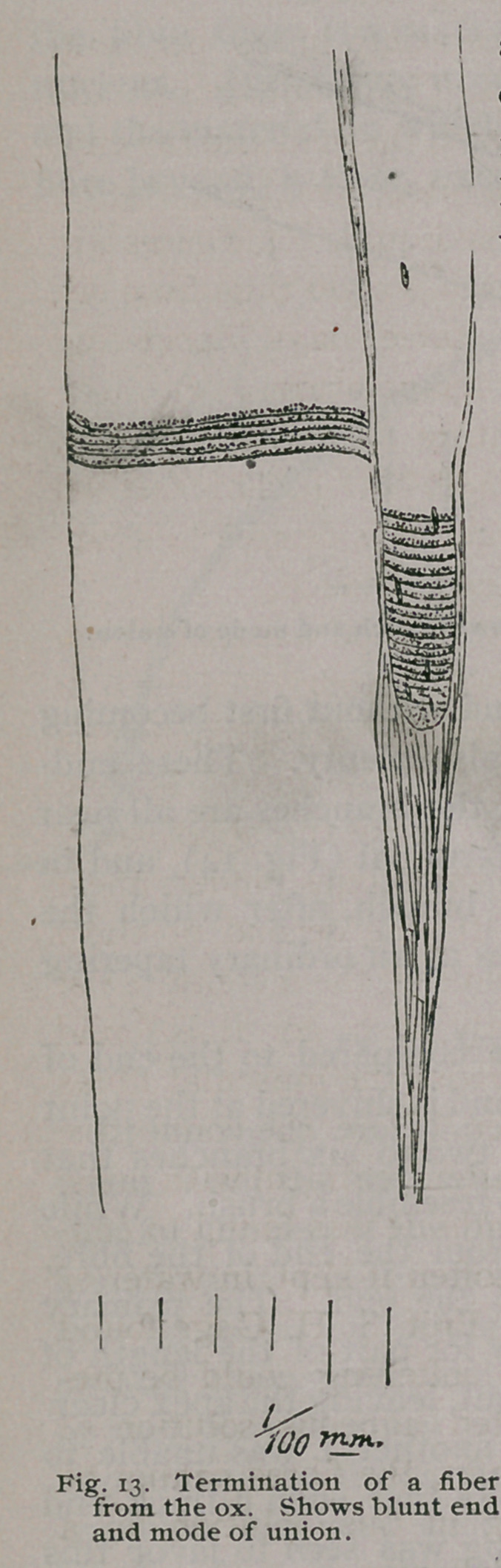


**Fig. 14. f10:**
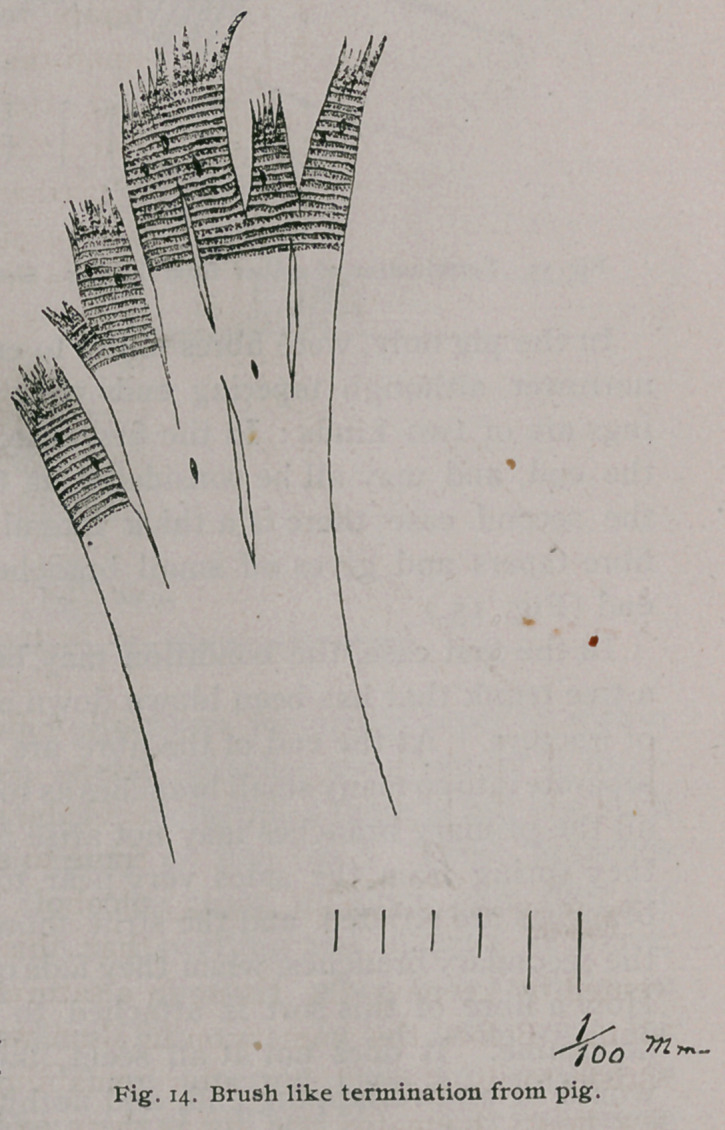


**Fig. 15. f11:**